# Interpersonal touch suppresses visual processing of aversive stimuli

**DOI:** 10.3389/fnhum.2015.00164

**Published:** 2015-04-08

**Authors:** Hiroaki Kawamichi, Ryo Kitada, Kazufumi Yoshihara, Haruka K. Takahashi, Norihiro Sadato

**Affiliations:** ^1^ Division of Cerebral Integration, Department of Cerebral Research, National Institute for Physiological Sciences, OkazakiJapan; ^2^ Graduate School of Human Health Sciences, Tokyo Metropolitan University, TokyoJapan; ^3^ School of Medicine, Faculty of Medicine, Gunma University, MaebashiJapan; ^4^ Department of Physiological Sciences, SOKENDAI (The Graduate University for Advanced Studies), HayamaJapan; ^5^ Department of Psychosomatic Medicine, Graduate School of Medical Sciences, Kyushu University, FukuokaJapan

**Keywords:** interpersonal touch, fMRI, attentional shift, aversive stimuli, visual cortex

## Abstract

Social contact is essential for survival in human society. A previous study demonstrated that interpersonal contact alleviates pain-related distress by suppressing the activity of its underlying neural network. One explanation for this is that attention is shifted from the cause of distress to interpersonal contact. To test this hypothesis, we conducted a functional MRI (fMRI) study wherein eight pairs of close female friends rated the aversiveness of aversive and non-aversive visual stimuli under two conditions: joining hands either with a rubber model (rubber-hand condition) or with a close friend (human-hand condition). Subsequently, participants rated the overall comfortableness of each condition. The rating result after fMRI indicated that participants experienced greater comfortableness during the human-hand compared to the rubber-hand condition, whereas aversiveness ratings during fMRI were comparable across conditions. The fMRI results showed that the two conditions commonly produced aversive-related activation in both sides of the visual cortex (including V1, V2, and V5). An interaction between aversiveness and hand type showed rubber-hand-specific activation for (aversive > non-aversive) in other visual areas (including V1, V2, V3, and V4v). The effect of interpersonal contact on the processing of aversive stimuli was negatively correlated with the increment of attentional focus to aversiveness measured by a pain-catastrophizing scale. These results suggest that interpersonal touch suppresses the processing of aversive visual stimuli in the occipital cortex. This effect covaried with aversiveness-insensitivity, such that aversive-insensitive individuals might require a lesser degree of attentional capture to aversive-stimulus processing. As joining hands did not influence the subjective ratings of aversiveness, interpersonal touch may operate by redirecting excessive attention away from aversive characteristics of the stimuli.

## Introduction

Interpersonal relationships serve a fundamental function for human health and well-being. Lack of interpersonal relationships, i.e., loneliness, is one of the major risk factors for health, along with smoking, or obesity (House et al., 1988a; Holt-Lunstad et al., 2010). Social support via interpersonal relationships for distressed others has a distress-alleviation regulatory function such as the suppression of painful thoughts and the repression of negative memories (Mikulincer et al., 2003).

Interpersonal touch is one type of social support. Physical contact with a person has been found to exert a stronger social support effect than verbal or emotional contact (Gallace and Spence, 2010). One striking example is that joining hands with a romantic partner suppresses brain activation during pain threat as compared to no physical contact (Coan et al., 2006). However, the neural mechanisms by which interpersonal touch suppresses brain activation accompanying distress are poorly understood.

Previous neuroimaging studies have shown that distress-related brain activation is suppressed by attending to cognitive tasks (Seminowicz et al., 2004) or paying attention to the sensation of breath (Zeidan et al., 2011). These findings raise the possibility that interpersonal touch suppresses distress-related activation by shifting attention from the cause of distress to interpersonal contact. If this is the case, we can assume that this suppression effect can be applied to processing of other stimuli. In the present study, we investigated the effect of interpersonal touch on the processing of aversive visual stimuli.

Previous neuroimaging studies have shown that observation of aversive stimuli such as others in distress produces activation in a distributed network of brain regions (Singer et al., 2004; Vollm et al., 2006; Akitsuki and Decety, 2009). This network includes regions of the visual cortex such as the lateral occipital area (BA 18 or 19; Lane et al., 1999; Mourao-Miranda et al., 2003; Singer et al., 2004; Vollm et al., 2006; Akitsuki and Decety, 2009) and limbic structures including the anterior cingulate cortex and anterior insula (Singer et al., 2004; Akitsuki and Decety, 2009). Activation in this network can be induced, because aversive stimuli attract more attention than non-aversive stimuli (Kawasaki et al., 2001; Vuilleumier, 2005; Pessoa, 2011). Indeed, a large body of literature demonstrates that the visual cortex is a major target of attentional modulation (Corbetta and Shulman, 2002). For instance, visual processing of objects in the occipito-temporal cortex is modulated by a range of factors, including painful stimuli (Bingel et al., 2007) and distractor stimuli present in cognitive tasks (Rose et al., 2005; Bingel et al., 2007; Klemen et al., 2009). Our attention is directed to the contact with others (e.g., contact with a familiar person; Stack and Muir, 1992; Peláez-Nogueras et al., 1996), which attenuates the negative response caused by aversive stimuli (Hertenstein, 2002). Accordingly, we can hypothesize that joining hands with a person can suppress activity of attention modulation target regions involved in the processing of aversive stimuli, such as the visual cortex. Moreover, increase of attentional resource toward aversive stimuli can depend on personality traits (Sullivan et al., 1995). Thus, personality traits can be associated with aversive-related brain responses, which are modulated by joining hands.

In the present study, we measured the brain activation of eight pairs of close friends (16 participants). During functional MRI (fMRI), participants completed a cognitive rating task related to aversiveness for aversive and non-aversive photographs. Participants completed the task under two conditions designed to manipulate social contact with others: (1) the human-hand condition, in which the participant placed their left hand on the left hand of their close friend; (2) the rubber-hand condition, in which the participants placed their left hand on a rubber hand. We predicted reduced activation of the visual cortex during the human-hand relative to rubber-hand condition. In addition, we predicted modulation of brain activity by an individual difference measure indexing sensitivity to aversive stimuli (pain-catastrophizing scale; PCS; Sullivan et al., 1995). Since individuals who have catastrophic thoughts about pain (i.e., persons with a higher PCS score) are more attentive to aversive stimuli (Sullivan et al., 1995, 2001), we predicted that the influence of social contact on the processing of aversive stimuli in the brain would differ depending on the PCS.

## Materials and Methods

### Participants

A total of eight pairs of female friends [aged 26.4 ± 2.5 years (mean ± SEM); minimum duration of friendship = 6 months (mean ± SEM = 72.0 ± 19.9 months)] took part in the experiment. We only recruited female participants, because we aimed to minimize cross-gender effects (e.g., sexual arousal) and because social support between friends of the same gender is particularly strong in females (House et al., 1988b). All participants had normal or corrected-to-normal visual acuity and were right-handed according to the Edinburgh handedness inventory (Oldfield, 1971). Participants were provided with monetary compensation. The protocol was approved by the ethical committee of the National Institute for Physiological Sciences, Okazaki, Japan. The experiments were undertaken in compliance with national legislation and the Code of Ethical Principles for Medical Research Involving Human Subjects of the World Medical Association (Declaration of Helsinki). All the participants provided written informed consent.

### Questionnaire

Participants completed two questionnaires. First, all participants completed the PCS (Sullivan et al., 1995). This 13-item scale measures the level of catastrophic thinking related to pain. Each item is evaluated using a five-point Likert scale (0, not at all; 1, to a slight degree; 2, to a moderate degree; 3, to a great degree; 4, all the time). Since the PCS measures catastrophizing about pain, which has been shown to result in more intense pain and emotional distress in response to pain (Sullivan et al., 1995, 2001), we used the total PCS score as a measure of trait pain sensitivity in order to index aversiveness sensitivity in the present task. The total PCS score was computed by summing the responses to all 13 items. Thus, the total PCS scores ranged from 0 to 52.

Second, participants rated the uncomfortableness they experienced during the human-hand condition and during the rubber-hand condition using two seven-point scales from 1 (not at all) to 7 (very uncomfortable). The questionnaires were completed after the fMRI experiment.

### Apparatus for Visual Presentation

Visual stimuli were presented using Presentation software 14.4 (Neurobehavioral Systems, Inc.) implemented on a personal computer (dc7900; Hewlett-Packard, Ltd.). A liquid crystal display (LCD) projector (CP-SX12000; Hitachi, Ltd.) located outside and behind the scanner projected the stimuli through a waveguide to a translucent screen, which the participants viewed via a mirror placed in the MRI scanner. The spatial resolution of the projector was 1,024 × 768 pixels, with a 60-Hz refresh rate. The distance between the screen and each participant’s eyes was approximately 175 cm, and the visual angle was 13.8 (horizontal) × 10.4 (vertical). Responses were collected via an optical button box (Current Designs, Inc.).

### Task Design

**FIGURE 1:**
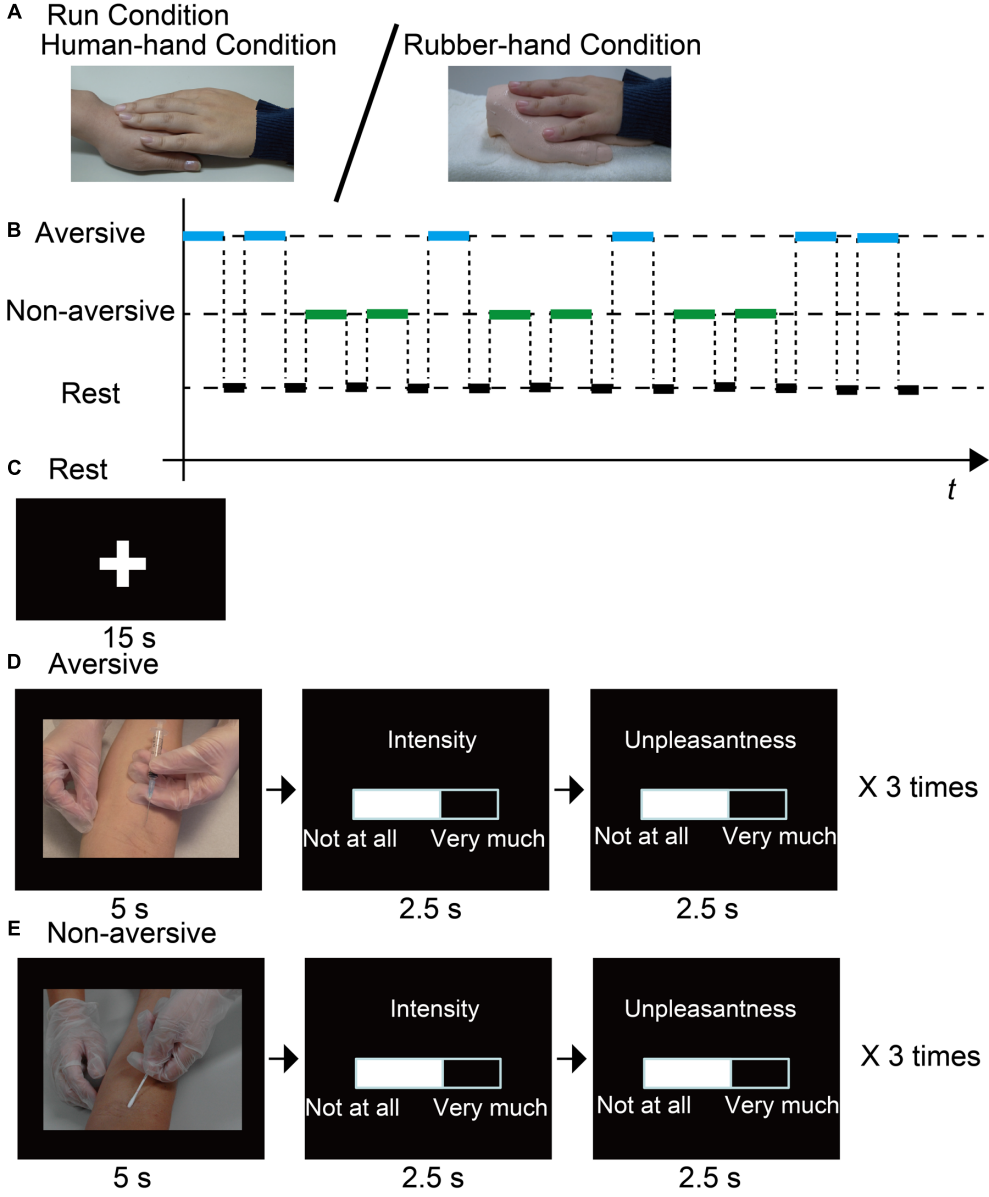
**Outline of experiment. (A)** Participants joined hands with a close friend or a rubber hand. **(B)** Example of a time chart of visual stimuli presentation sequence. **(C)** In between stimulus blocks, a fixation cross was presented at the center of the screen for 15 s. **(D,E)** During target blocks (30 s), participants were presented with three aversive or non-aversive visual stimuli (5 s). For each stimulus, participants rated unpleasantness and pain intensity using a visual analog scale (5 s).

The task consisted of three runs. In two of the runs, participants were shown aversive and non-aversive photographs. For each photograph, participants evaluated the intensity of pain and degree of unpleasantness felt by the person shown in the photograph (aversive-evaluation runs). The two aversive-evaluation runs differed in touch: the participant’s left hand was placed either on the left hand of their partner (human-hand run) or on a rubber hand (rubber-hand run) (**Figure 1A**). The rubber hand was produced from the cast of an adult’s left hand with gender-neutral features (see Kitada et al., 2010 for details of the production of the rubber hand). In the remaining run, the participants were seated outside the scanner with their left hand supporting the left hand of their partner, while their partner was evaluating the aversiveness of visual stimuli in their own human-hand run (lending-hand run). The lending-hand run was conducted before or after the two aversive-evaluation runs, with the order counterbalanced between participants. Likewise, the order of the human-hand and rubber-hand runs was counterbalanced in the aversive-evaluation runs between participants.

We employed a conventional block design. Each aversive-evaluation run involved six blocks for the evaluation of aversive stimuli (aversive block) and six blocks for the evaluation of non-aversive stimuli (non-aversive block). Each block lasted for 30 s, followed by a 15-s rest block to allow the blood-oxygen-level dependent (BOLD) signal to return to baseline levels between task blocks (30-s block + 15-s rest × 12 = 540 s, **Figures 1B,c**). We presented three stimuli during each task block [3 stimuli × 6 blocks × 2 types (aversive/non-aversive) = 36 stimuli in total]. While each visual stimulus was presented for 5 s, the participant was instructed to imagine the pain intensity and unpleasantness experienced by the person shown in the photograph. After the 5-s presentation of the stimulus, the participant reported the estimated pain intensity and unpleasantness using a visual analog scale (VAS) ranging from 0 (not at all painful or unpleasant) to 100 (extremely painful or unpleasant) using the right index and middle fingers. Pushing the button with the right index finger decreased the value of the VAS, whereas pushing the button with the right middle finger increased the value of the VAS. The amount of change of the value of the VAS was dependent on the time of pressing the button. Quitting the button press stopped the value change of the VAS. After setting the appropriate value, participants were required to wait until the end of each rating phase (2.5 s) to validate the value of the VAS. The order of reporting pain intensity and unpleasantness was counterbalanced within each run. Durations for reporting pain intensity and unpleasantness were 2.5 s.

The aversive visual stimuli were scenes showing an injection to the arm or foot ( **Figure 1D**), whereas the non-aversive visual stimuli were scenes showing a Q-tip being pressed to the arm or foot ( **Figure 1E**). The compositions (e.g., number of persons, body part, and positional relation) between the two types of stimuli were highly similar. Visual stimuli were selected from a set of 24 stimulus pairs consisting of aversive and non-aversive photographs with similar compositions. The 24 pairs were rated on pain intensity and aversiveness by nine independent raters [aged 30.9 ± 2.3 years (average ± SEM); five male], who were blind to the purpose of the present study. Based on the rating results, a set of 18 pairs of photographs was selected for the present fMRI study, such that the pain intensity and unpleasantness differed significantly between the aversive and non-aversive photographs [for pain intensity* p* < 0.001 (average score (±SEM) aversive, 74.8 (±1.1), non-aversive, 8.7 (±0.9)); for unpleasantness *p* < 0.001 (aversive, 72.6 (±1.1), non-aversive, 38.9 (±1.4))].

### fMRI Data Acquisition

A 3-T scanner (Verio; Siemens, Ltd., Erlangen) was used for MRI. The participant’s head was immobilized within a 32-element phased-array head coil. fMRI was performed using an echo planar imaging (EPI) gradient-echo sequence [echo time (TE) = 30 ms; repetition time (TR) = 2,500 ms; field of view (FOV) = 192 mm × 192 mm; flip angle = 80^∘^; matrix size = 64 × 64; 39 slices; slice thickness = 3 mm; and total number of volumes = 220]. A whole-brain high-resolution, T1-weighted anatomical MR image using magnetization prepared rapid acquisition gradient echo (MP-RAGE) was also acquired for each participant (TE = 2.97 ms; TR = 1,800 ms; FOV = 256 mm × 256 mm; flip angle = 9^∘^; matrix size = 256 × 256 pixels; and slice thickness = 1 mm).

### fMRI Data Analysis

A single participant reported the same values for each of the aversive and non-aversive stimuli, and was therefore judged to have misunderstood the task instructions. Data from this participant were excluded. The final data set consisted of the findings from 15 participants.

We used SPM8 revision 4667 (Wellcome Trust Centre for Neuroimaging; http://www.fil.ion.ucl.ac.uk/spm; Friston et al., 2007) in MATLAB 2011b (MathWorks, Inc.) to analyze the functional images. The first four volumes of each fMRI run were discarded to allow for T1 equilibrium effects. To correct subject’s head motion, functional images from each run were realigned to the first image, and again realigned to the mean image after the first realignment. After the realignment processes, we checked head-movement parameters. None of the runs included head movements over 3 mm. We corrected slice timing within each image to the middle slice by applying Fourier phase-shift interpolation. Then, the mean of the realigned EPI images was co-registered with the T1-weighted MP-RAGE image. Subsequently, the co-registered T1-weighted MP-RAGE image was normalized to the Montreal Neurological Institute (MNI) template, involving linear and nonlinear three-dimensional (3D) transformations. The parameters from this normalization process were applied to each of the EPI images. Finally, the anatomically normalized EPI images were resampled to a voxel size of 2 mm × 2 mm × 2 mm and spatially smoothed using a Gaussian kernel of 8 mm full width at half maximum (FWHM; final smoothness values: *x* = 11.8 mm; *y* = 11.8 mm; *z* = 11.9 mm).

Task-related activation was statistically evaluated using the general linear model (GLM) at the individual level to generate contrast images, which in turn were incorporated into random-effects analysis at the group level (Friston et al., 1994). In the individual level analysis, we defined four regressors (rubber-hand aversive, rubber-hand non-aversive, human-hand aversive, and human-hand non-aversive); a further six regressors represented head movements (realignment parameters). Participants needed to remember the aversiveness of stimuli when rating their pain intensity and unpleasantness. As an aversive response can be caused both when viewing and when recalling aversive stimuli, we modeled all four regressors for the six 30-s blocks, including the period when participants rated their pain intensity and unpleasantness (5 s). As each regressor contains the response-related phase, the irrelevant components of the rating phase (e.g., moving the VAS) should be canceled out in the comparison between the four conditions.

After the individual level analyses were completed, we conducted group-level analysis using the contrast images produced by the individual level analysis [contrasts of common aversive effects (aversive (rubber-hand + human-hand) > non-aversive (rubber-hand + human-hand)) and interaction effects between rubber/human hand effects and aversive effects (rubber-hand (aversive – non-aversive) > human-hand (aversive – non-aversive))]. To examine the main effects of hand type, we also compared the rubber-hand (aversive + non-aversive) with human-hand (aversive + non-aversive) conditions. The statistical threshold for these analyses was set at *p <*0.005 uncorrected at the peak level, and *p <*0.05 at the cluster level family-wise error (FWE) corrected over the whole brain. In terms of significant activation related to the interaction effects between rubber/human hand effects and aversive effects, the average beta value (parameter estimate) of the four conditions (rubber-hand aversive, rubber-hand non-aversive, human-hand aversive, and human-hand non-aversive) within a 12-mm diameter sphere located at the peak (*x* = 26, *y* = –80, *z* = 0) of a significant cluster were calculated for 15 subjects. The diameter was defined according to the final smoothness. A spherical region of interest (ROI) was defined for the following analysis because the beta value around the peak represented the characteristics of the significant cluster well. We then conducted *post hoc* analysis to compare the beta values of the four conditions. Anatomical labeling of the activated clusters was performed using the Anatomy toolbox v1.8 (Eickhoff et al., 2005).

Using the results of the fMRI analysis, we conducted simple regression analysis between the total PCS and the average beta value of (rubber-hand aversive > human-hand aversive) within a 12-mm diameter sphere located at the peak coordinates (*x* = 26, *y* = –80, *z* = 0) of a group-level significant cluster related to the interaction between hand effect and aversive effect.

### Behavioral Data Analysis

We conducted statistical analysis of the rated pain intensity and unpleasantness. This involved two two-way [aversive (aversive/non-aversive) × hand condition (human/rubber)] repeated measures analyses of variance (rmANOVA) on pain intensity and unpleasantness ratings.

## Results

### Questionnaire Results

**FIGURE 2:**
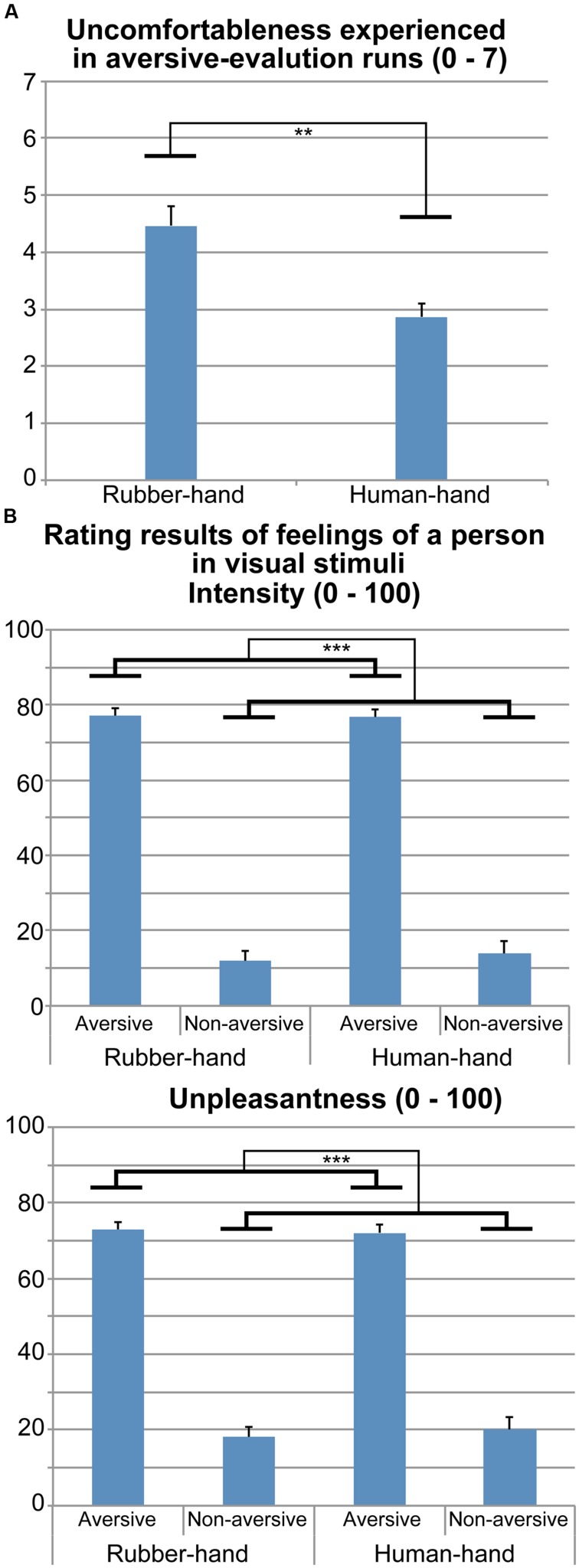
**Rating results during and after fMRI experiment.** **(A)** Uncomfortableness experienced during the two aversive-evaluation runs (human-hand and rubber-hand runs) is shown. Contact with the rubber hand was more unpleasant than contact with the human hand (paired-*t* test, *p* < 0.01). **(B)** Rating results regarding pain intensity and unpleasantness that the person depicted in the photograph stimuli would feel. Pain intensity ratings for rubber-hand aversive, rubber-hand non-aversive, human-hand aversive, and human-hand non-aversive stimuli were 77.0 (±2.1), 11.9 (±2.6), 76.7 (±2.1), and 13.8 (±3.5), respectively. Unpleasantness ratings for rubber-hand aversive, rubber-hand non-aversive, human-hand aversive, and human-hand non-aversive stimuli were 72.9 (±2.9), 18.2 (±3.6), 72.0 (±3.6), and 20.0 (±4.8), respectively. Two separate two-way rmANOVAs (hand × aversiveness) on pain intensity and unpleasantness ratings showed significant main effects of aversiveness (*p-*values* <*0.001). **p < 0.01, ***p < 0.001.

The mean (±SEM) score on the PCS was 24.6 (±2.5). The mean (±SEM) uncomfortableness ratings during the human- and rubber-hand conditions were 2.9 (±0.2) and 4.5 (±0.4), respectively. The paired samples *t*-test showed a significant difference in uncomfortableness ratings between the human- and rubber-hand conditions [*t*(14) = 3.36, *p <*0.01; **Figure 2A**].

### Rating Results: Pain and Unpleasantness Ratings during fMRI

**Figure 2B** shows the pain intensity and unpleasantness of aversive stimuli in the fMRI experiment. Two separate two-way rmANOVA [aversiveness (aversive/non-aversive) × hand condition (human/rubber)] on pain intensity and unpleasantness ratings showed main effects of aversiveness: there were greater ratings for aversive than non-aversive stimuli [pain intensity, *F*(1,14) = 255.45, *p <*0.001; unpleasantness, *F*(1,14) = 67.30, *p <*0.001]. We did not find any significant main effects of hand [pain intensity, *F*(1,14) = 0.37, *p* = 0.554; unpleasantness, *F*(1,14) = 0.07, *p*= 0.791] and interaction effects [pain intensity, *F*(1,14) = 1.06, *p* = 0.320; unpleasantness, *F*(1,14) = 1.14, *p* = 0.303].

### fMRI Results

**FIGURE 3:**
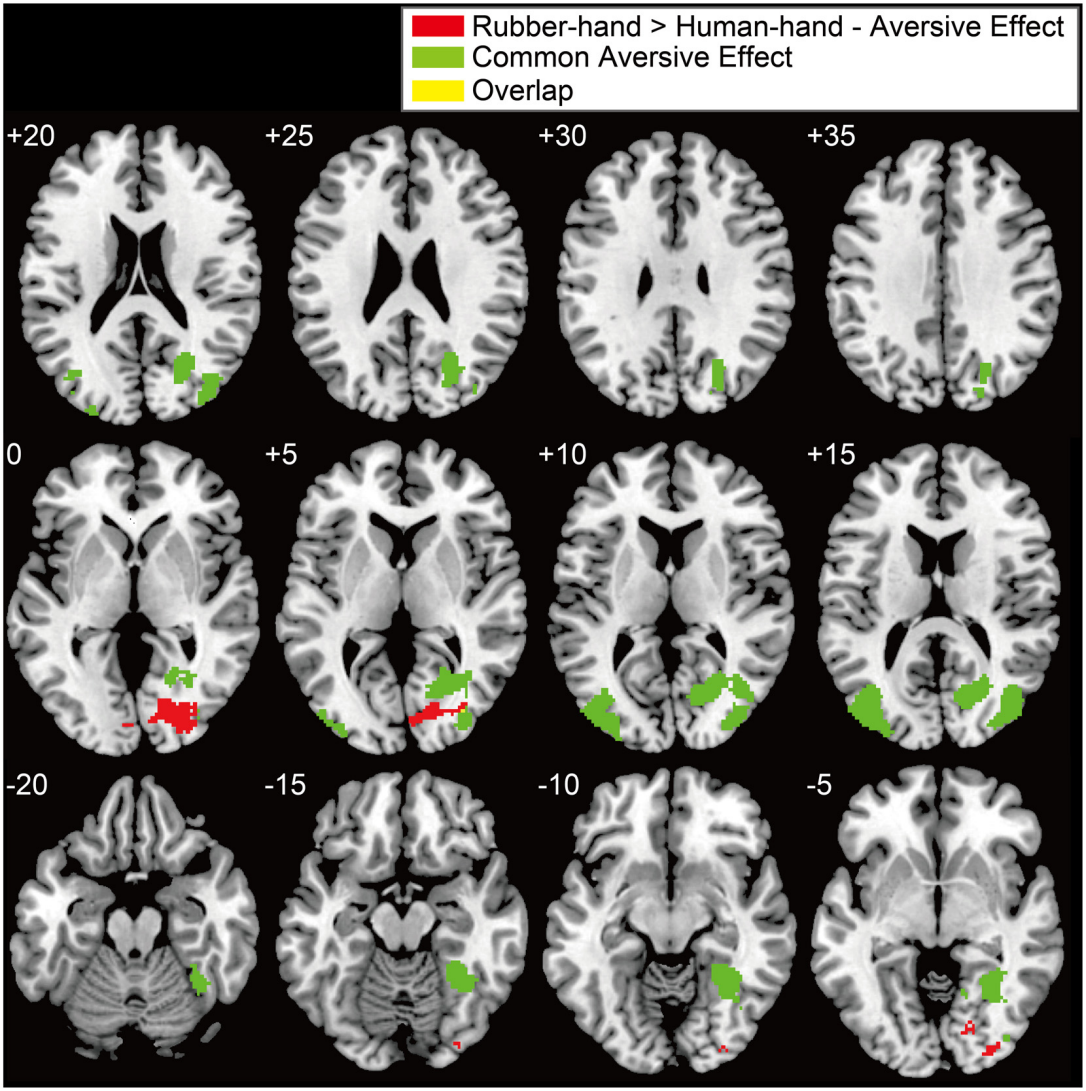
**Significant activation for the interaction between hand and aversiveness conditions (rubber-hand > human-hand - aversive effects) and common aversive effects.** Red, green, and yellow clusters showed interaction effects, common aversive effects, and overlapped clusters, respectively. Threshold of these activations was at peak level uncorrected *p* < 0.005 and at cluster level family-wise error (FWE) corrected *p* < 0.05. The left upper number of each section indicates z coordinates.

Aversive effects that were common to both the human- and rubber-hand conditions (common aversive effects) showed significant activations in both visual areas [two clusters; peak voxels = (*x* = 26, *y* = - 64, *z* = 22) and (*x* = - 36, *y* = - 70, *z* = 18); **Table 1**; **Figure 3** green-colored area]. On the other hand, we observed no main effect of hand type; neither the contrast of [human-hand (aversive + non-aversive) > rubber-hand (aversive + non-aversive)] nor [rubber-hand (aversive + non-aversive) > human-hand (aversive + non-aversive)] revealed any significant activation.

**Table 1:** Significant activation for common aversive effects [aversive (rubber-hand + human-hand) > non-aversive (rubber-hand + human-hand)].

	Cluster *p*(FWE)	*x*	*y*	*z*	Cluster size (number of voxels)	*t*-value
Right visual area	<0.001	26	-64	22	2050	5.78
Left visual area	0.015	-36	-70	18	556	5.49


*The threshold of these activations was at peak level uncorrected *p*< 0.005 and at cluster level family-wise error (FWE) corrected *p* < 0.05.*

**FIGURE 4:**
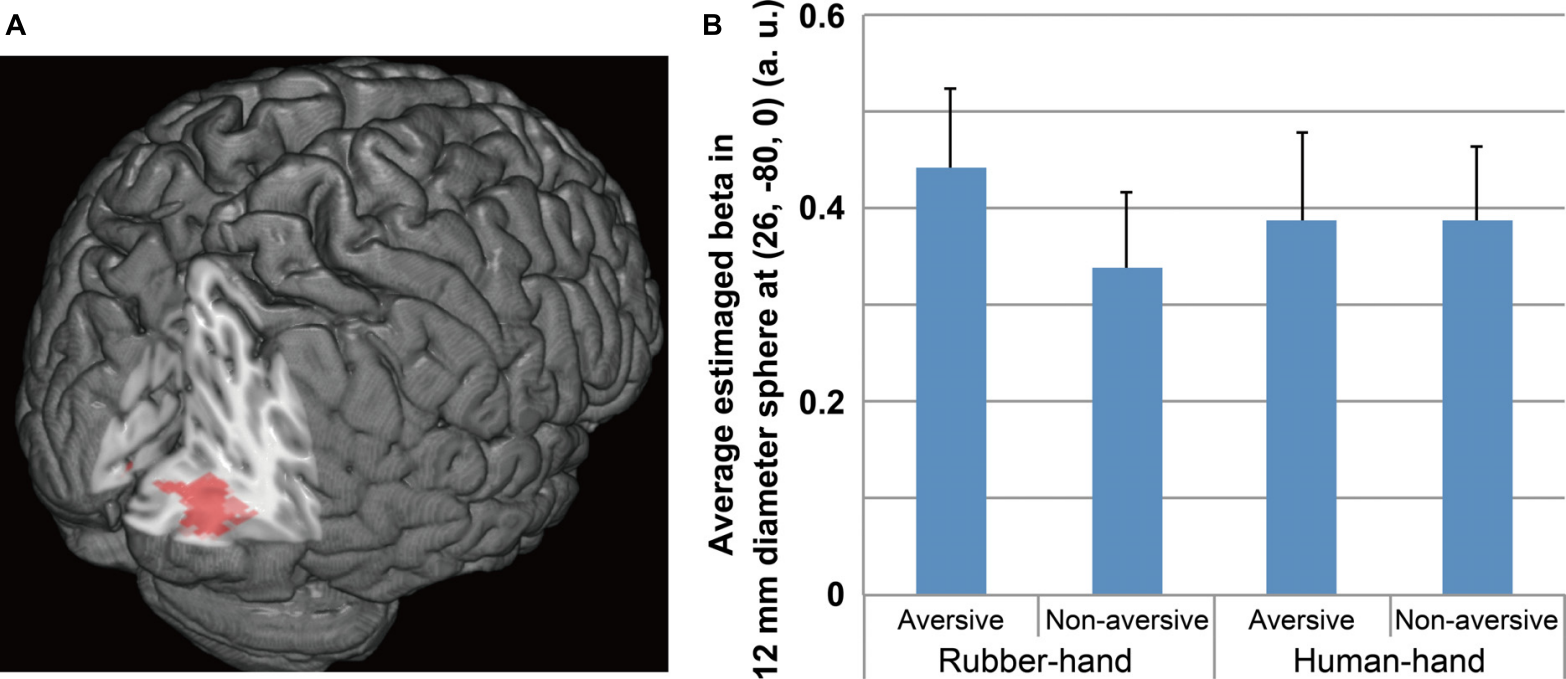
**Significant activation for the interaction between hand and aversiveness conditions.** **(A)** Significant cluster is shown. The peak of the significant cluster is located at the section. **(B)** Average estimated beta of each condition in 12 mm diameter sphere at (26, –80, 0) is shown. The threshold of activation at peak level uncorrected *p* < 0.005 and at cluster level FWE corrected *p* < 0.05. *Post hoc* analysis showed that the aversive effect in the rubber-hand condition significantly differed (Bonferroni corrected *p* < 0.05), whereas that in the human-hand condition did not.

Regions of significant activation were observed for the interaction between hand (human-hand vs. rubber-hand) and aversiveness (aversive vs. non-aversive) in the right visual areas [peak voxel = (*x* = 26, *y* = - 80, *z* = 0); **Table 2**; **Figure 3** red-colored area and **Figure 4**]. In addition, regions of activation revealed by these two contrasts (the common effects related to aversiveness and the interaction between hand type and aversiveness) showed little overlap (yellow-colored area in **Figure 3**; section of z coordinates = 5). Furthermore, even with a more lenient peak threshold (uncorrected *p* < 0.01) for the common aversive effects, there was little overlap [39 voxels (8.9%)] between these two contrasts. *Post hoc* analysis showed that only the comparison between the rubber-hand aversive and rubber-hand non-aversive conditions showed a significant difference (Bonferroni corrected *p* < 0.05, **Figure 4B**).

**Table 2:** Significant activation for the interaction between hand and aversiveness [rubber-hand (aversive – non-aversive) > human-hand (aversive – non-aversive)].

	Cluster *p*(FWE)	*x*	*y*	*z*	Cluster size (number of voxels)	*t*-value
Right visual area	0.027	26	-80	0	436	5.17

*The threshold of these activations was at peak level uncorrected *p*< 0.005 and at cluster level FWE corrected *p* < 0.05.*

We analyzed the cluster of activation in the visual cortex according to the anatomical probabilistic map (Eickhoff et al., 2005). The cluster of the interaction effects consisted of regions within V1, V2, V3v, and V4. By contrast, two clusters of visual areas found in common aversive effects included neither V3v nor V4. Instead, these clusters included regions of activation within V1 and V5 (**Table 3**).

**Table 3:** Classifications of the visual areas (V1, V2, V3v, V4, and V5) in three clusters.

	Cluster size (number of voxels)	V1	V2	V3v	V4v	V5
**Aversive (rubber-hand + human-hand) > non-aversive (rubber-hand + human-hand)**
Right visual area (26, -64, 22)	2050	396 (17.2%)	31 (1.9%)	-	-	10 (10.5%)
Left visual area (-36, -70, 18)	556	-	-	-	-	47 (65.0%)
**Rubber-hand (aversive – non-aversive) > human-hand (aversive – non-aversive)**
Right visual area (26, -80, 0)	436	44 (1.8%)	78 (4.4%)	44 (1.8%)	59 (9.4%)	-

*Anatomical labeling of the activated clusters was performed using the Anatomy toolbox v1.8 (Eickhoff et al., 2005). The number of activated voxels and % of activated voxels in each visual area are shown.*

**FIGURE 5:**
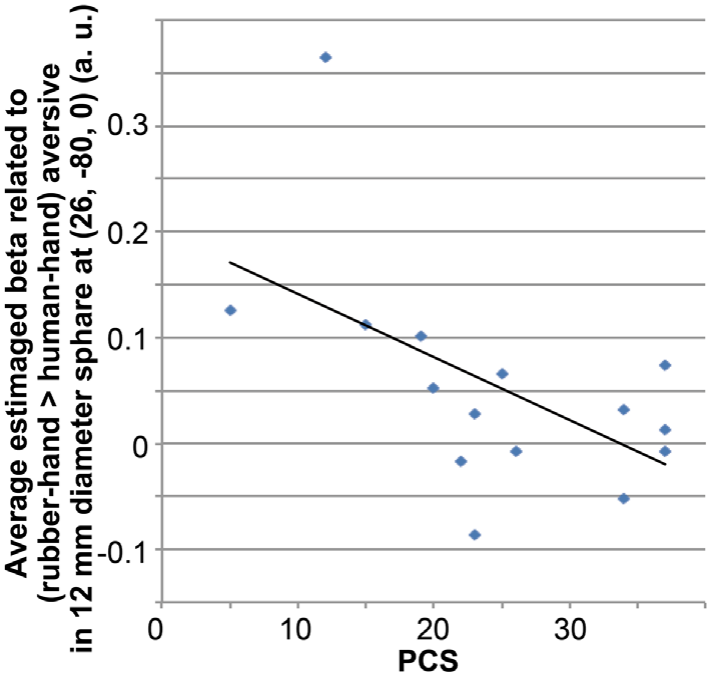
**Correlation between average beta value related to (rubber-hand > human-hand) aversive condition and total PCS score.** Average beta value was calculated within a sphere of 12-mm diameter located at the peak of significant activation from the interaction between hand effects and aversive effects.

Simple regression analysis between the total PCS scores and estimated beta values fromthe (rubber-hand – human-hand) aversive contrast revealed a significant negative correlation (*R* = –0.556; *p* = 0.031; **Figure 5**).

## Discussion

### Effects of Aversiveness on Behavioral Results

Participants gave unpleasantness and simulated pain intensity ratings for aversive and non-aversive photographic stimuli. The results showed significantly higher unpleasantness and pain intensity ratings for aversive than non-aversive visual stimuli. These results suggest that the participants cognitively recognized the aversiveness of the visual stimuli.

### Effects of Aversiveness on fMRI Results

Compared with non-aversive visual stimuli, greater activation was found in visual cortical areas for aversive visual stimuli during the rubber- and human-hand conditions. In this sense, the present results support the previous studies showing aversiveness-related activation in visual cortical areas (Lane et al., 1999; Mourao-Miranda et al., 2003). As the aversive-related activation in the present study partially overlapped with the visually body-sensitive activation found in our previous studies (3–15% in the right cluster, and 21–47% in the left cluster; Kitada et al., 2014; Okamoto et al., 2014), the common aversive effects might involve body-related aversive processing such as physical pain. Thus, aversive characteristics (pain intensity and unpleasantness) are associated with increased visual cortical activation.

### Behavioral Effect of Joining Hands with a Person

The results of a questionnaire measuring comfortableness completed after the fMRI session indicated that joining hands with a person was more comfortable than joining with a rubber hand. Since the set of visual stimuli was identical across the human- and rubber-hand conditions, and the order of the two conditions was counterbalanced among the participants, the current comfortableness rating results can be attributed to the target of the joined hands. This result can be partly explained by the uncanny valley effect (Mori, 1970). In this effect, humans have an unpleasant reaction toward an almost perfectly realistic human model (Mori, 1970) and its abnormal features (Seyama and Nagayama, 2007). In this experiment, the rubber and real hands looked highly similar, whereas the rubber hand alone was disconnected from the forearm. Thus, the uncanny valley effect may contribute to the more uncomfortable feeling related to joining hands with the rubber hand compared with joining hands with a friend. In addition to this effect, it is known that photographic or video stimuli showing participants’ romantic partner or child activates the reward system (Bartels and Zeki, 2000, 2004; Aron et al., 2005; Noriuchi et al., 2008; Xu et al., 2011a; Acevedo et al., 2012). In this sense, the presence of a familiar person might be experienced as rewarding. In addition, since humans communicate emotional information through touch (Fisher et al., 1976; Whitcher and Fisher, 1979; Hertenstein et al., 2006; Lederman et al., 2007; Kitada et al., 2013), joining hands with a person, especially with a familiar person, might give rise to positive affect.

### Modulation of Visual Processing Caused by Joining Hands with a Person

The interaction between hand (human-hand vs. rubber-hand) and aversiveness condition [rubber-hand (aversive > non-aversive) – human-hand (aversive > non-aversive)] was significant in the right visual cortex. Furthermore, the rubber-hand aversive condition showed significantly greater activation than the rubber-hand non-aversive condition, whereas the human-hand aversive condition did not show significantly greater activation than the human-hand non-aversive condition. To the best of our knowledge, this is the first demonstration that the neural response to aversive stimuli in the visual cortex can be suppressed by social contact through interpersonal touch. Consistent with previous studies showing that aversive (unpleasant and high arousal) visual stimuli cause activation in visual areas (Lane et al., 1999; Mourao-Miranda et al., 2003), aversive visual stimuli during the rubber-hand condition activated visual perception areas (V2, V3v, and V4). Since the response in V2 and V4 can be modulated by top–down attention (Kastner and Ungerleider, 2000), one possible explanation for this interaction effect is that the effect of aversiveness during the human-hand condition was suppressed via an attentional shift away from aversive visual processing and toward the processing of interpersonal touch. Furthermore, the location of visual cortical activation in the current study overlaps with that shown in a previous study indicating that attentional modulation affects visual object processing in the lateral occipital complex (Rose et al., 2005; Bingel et al., 2007; Klemen et al., 2009) via modulation by higher visual perceptual areas including V4 (Rose et al., 2005). The present result is consistent with the suggestion that attention to positive emotional information via interpersonal touch has a modulatory effect on visual perceptual processing.

Simple regression analysis showed a negative correlation between total PCS score and average activation around the peak of the cluster of interaction effects related to rubber-hand aversive > human-hand aversive. Participants scoring more highly on the PCS scale are more sensitive to aversive visual stimuli in comparison with participants scoring lower on the PCS scale (Sullivan et al., 1995, 2001). This sensitivity to aversive stimuli may recruit attentional focus such as recursive thoughts about aversive visual stimuli (Sullivan et al., 1995), and this might lead to a requirement for a higher load of information processing in the visual cortex. Thus, the present negative correlation results suggest that participants who are more sensitive to aversiveness show a reduced tendency to show the reported effect of a familiar friend’s hand, due to a higher processing load caused by aversive stimuli.

The brain areas showing the interaction between hand type and aversiveness ratings (V2, V3v, and V4) are located in the ventral visual pathway (Rottschy et al., 2007; Wilms et al., 2010), also known as the ‘what’ pathway in visual perception (Ungerleider and Mishkin, 1982; Goodale and Milner, 1992). Ventral occipital lesions impair explicit discrimination of objects’ properties, although patients are still able to reach and grasp the objects (James et al., 2003). Thus, the present result showing modulation of visual processing during the human-hand condition may be implemented via suppression of the processing of object features. Joining hands with a person may suppress feature inspection for aversive visual stimuli.

The two aversive rating results (unpleasantness and pain intensity) did not show hand effects. This suggests that cognitive evaluation of visual stimuli was similar during human- and rubber-hand conditions. In addition, there was little overlap of the activation in visual cortex between the aversive effects specific to the rubber-hand and the common aversive effects. As processing of the cognitive evaluation of aversiveness should be reflected in the activation related to the common aversive effects, this result indicates that the visual cortical region showing aversive-related activation specific to the rubber-hand (**Figure 3**) was not engaged in the cognitive evaluation process. Thus, aversive-related activation in the rubber-hand condition might be redundant visual processing. Furthermore, we found no overlap between the present rubber-hand-specific activation and that reported in visually body-sensitive areas in previous studies (Kitada et al., 2014; Okamoto et al., 2014). Based on these results, we speculated that the modulation target process was not restricted to body-related processing (e.g., physical pain or interpersonal touch), and that it might include more general processes for redundant processing or rumination (e.g., visual imagery related to negative feeling). Rumination about aversive stimuli can lead to negative thoughts (Sullivan et al., 1995). We suggest that joining hands with a person suppress redundant visual processing, thus, leading to reduced stress or negative emotion. In this sense, joining hands with a person might have a security-provision function through attention modulation, which is afforded by social relationships (Mikulincer et al., 2003).

Based on these results and previous findings, we hypothesize how these interpersonal touch effects are related to attention modulation as follows. Interpersonal touch evokes positive feelings, which can suppress negative emotions (e.g., emotions aroused by aversive stimuli; Hertenstein, 2002). Furthermore, interpersonal touch contributes to social connections (Gallace and Spence, 2010) and social bonding (Dunbar, 2010) via various mechanisms, including psychopharmacological ones (e.g., release of neuropeptides such as oxytocin and endorphins; Dunbar, 2010). As humans have a fundamental motivation to form positive social relationships (Baumeister and Leary, 1995), interpersonal touch captures our attention because it reminds us of social relationships. In order to further investigate these mechanisms, future studies should clarify whether other factors involved in interpersonal touch, such as the social closeness of the person (e.g., familiar vs. unfamiliar person) and the object properties of a human skin (e.g., shape, warmth, and smoothness of one’s hand), are critical modulators of the response toward aversive stimuli.

### Limitations

In the present study, we did not find empathy-related brain activation in the pain matrix, including limbic structures (Hutchison et al., 1999; Singer et al., 2004, 2006; Cheng et al., 2010). Empathic brain responses are modulated by various kinds of relationships and by participants’ personality features (de Vignemont and Singer, 2006). One of the major factors that leads to enhanced empathic brain responses is familiarity (Singer et al., 2004; Cheng et al., 2010; Kawamichi et al., 2013). Furthermore, the aversive response to visual stimuli showing an injection to the arm or foot (similar to in the present study) could be suppressed by the context [e.g., suppression of the affective link caused by a professional relationship between the participant and the target (Decety et al., 2010), and association with a desired outcome (Lamm et al., 2007)]. The lack of empathy-related pain-matrix activation in our study may be partly due to the fact that an unfamiliar person without affective link, rather than a familiar person, was receiving aversive visual stimuli. However, the aim of the present study was to investigate the modulatory effects of joining hands on visual perceptual processing. Since low processing load is necessary for investigating attention modulation effects (Xu et al., 2011b), using aversive visual stimuli showing unfamiliar others is preferable, as this will be less emotionally arousing. This suggests that, in studies designed to investigate modulatory effects on visual perceptual processing, absence of pain-related empathic brain responses is to be expected.

## Conclusion

Joining hands with a person arouses positive feelings. The current study showed evidence that the interpersonal touch effect caused by joining hands with a person suppresses visual perceptual processing in visual cortical areas, in a way that is similar to attention modulation processes. This hand-modulated visual activation was located in areas including V1, V2, V3, and V4v, which differed from the location (in areas including V1, V2, and V5) of the aversive-related activation commonly caused by the two conditions (i.e., rubber-hand and human-hand). This effect was particularly evident for more aversiveness-insensitive participants, who might allocate less attentional resources to processing aversive stimuli. We conclude that interpersonal touch prevents redundant visual inspection of aversive stimuli.

## Author Contributions

HK designed the experiments, conducted the experiments, analyzed the fMRI data, and wrote the manuscript. RK designed and conducted the experiments, and wrote the manuscript. KY participated in experimental design and conducted the experiments. HKT conducted the experiments. NS supervised the overall project and edited the manuscript.

## Conflict of Interest Statement

The authors declare that the research was conducted in the absence of any commercial or financial relationships that could be construed as a potential conflict of interest.
